# Framing the decision to contract out elderly care and primary health care services – perspectives of local level politicians and civil servants in Finland

**DOI:** 10.1186/1472-6963-12-201

**Published:** 2012-07-17

**Authors:** Liina-Kaisa Tynkkynen, Juhani Lehto, Sari Miettinen

**Affiliations:** 1 School of Health Sciences, University of Tampere, Tampere, Finland

## Abstract

**Background:**

In the literature there are only few empirical studies that analyse the decision makers’ reasoning to contract out health care and social services to private sector. However, the decisions on the delivery patterns of health care and social services are considered to be of great importance as they have a potential to influence citizens’ access to services and even affect their health. This study contributes to filling this cap by exploring the frames used by Finnish local authorities as they talk about contracting out of primary health care and elderly care services. Contracting with the private sector has gained increasing popularity, in Finland, during the past decade, as a practise of organising health care and social services.

**Methods:**

Interview data drawn from six municipalities through thematic group interviews were used. The data were analysed applying frame analysis in order to reveal the underlying reasoning for the decisions.

**Results:**

Five argumentation frames were found: *Rational reasoning; Pragmatic realism; Promoting diversity among providers; Good for the municipality; Good for the local people*. The interviewees saw contracting with the private sector mostly as a means to improve the performance of public providers, to improve service quality and efficiency and to boost the local economy. The decisions to contract out were mainly argued through the good for the municipal administration, political and ideological commitments, available resources and existing institutions.

**Conclusions:**

This study suggests that the policy makers use a number of grounds to justify their decisions on contracting out. Most of the arguments were related to the benefits of the municipality rather than on what is best for the local people. The citizens were offered the role of active consumers who are willing to purchase services also out-of-pocket. This development has a potential to endanger the affordability of the services and lead to undermining some of the traditional principles of the Nordic welfare state.

## Background

This paper addresses the types of framings used by local Finnish authorities when they argue about contracting out primary health care and elderly care services to the private sector. Thus, in this paper contracting out is addressed in the context of a health care systems mainly based on so called Beverigian model, i.e. on tax-funding and the dominance of public providers. By contracting we mean a relationship between a public purchaser and a private, not-for-profit or for-profit providers that engage in a contractual relationship in order to deliver public services [1]. The selection of the private providers usually involves a process of competitive bidding organised by the public sector. We also include vouchers in this definition because in Finland the providers eligible for delivering services purchased by vouchers, are selected by the municipality via competitive bidding. In general, contracting here refers to a notion according to which the public sector retains the main responsibility for financing and regulating the services as well as for monitoring the performance of service providers [2]. We acknowledge that there are also other terms, such as outsourcing and privatization of the provision, referring to similar activities. We use the term contracting out to refer to all these activities throughout the paper.

Contracting out public services has gained substantial popularity in several countries such as the Nordic countries, United Kingdom, New Zealand, Australia and Canada e.g.[2-5]. The developments towards increasing privatization in the welfare have been described as a long historical process reflecting the broad transformations in the western societies influenced by economic and cultural changes and policy diffusion [6]. These societal changes have been suggested to create an environment in which the public-private boundaries may start to melt [7] and through which health care and social service systems might become more private in nature [3]. It has indeed been argued that, during the past decades, health care and social service systems in Europe have incrementally started to shift towards private provision, financing, management and investments [7]. In spite of this, not much literature exists concerning the motivations that drive the decisions to contract with the private sector in health care and social services see, however, e.g. [8-11]. The decisions on the delivery patterns may be seen, however, to be of great importance as they have a potential to influence citizens’ access to services and even affect their health [12].

The aim of this study is to explore the argumentation frames used by local politicians and civil servants when they argue about contracting out health care and social services. In order to do this we employed frame analysis initially introduced by Goffman [13]. The framing of the policy problems creates rationales that authorize some policy solutions and not others [14]. Thus, frame analysis provides a tool for uncovering the underlying beliefs, perceptions and appreciations of policy makers [15].

The study is based on interview data collected through thematic interviews in six municipalities in Finland. The interviewees include civil servants responsible for purchasing health and social services and elected officials responsible for setting the annual budget for health and social care and for the political decisions on purchasing services from private providers. The analysis resulted in five analytical frames. The main emphasis in the frames was on the benefits for the municipality rather than on the good of the local people.

The article proceeds as follows: In the next section we briefly review the literature on contracting out of public services in different contexts. After that we describe the purpose of the study, methods, study context and the data in detail. Finally, we present the results of the analysis and discuss their significance. We conclude by summarising the main results of the study.

### Why to contract out and why not?

Public, for-profit and not-for-profit have been assumed to pursue different societal goals [16,17] and potentially to possess certain qualities that make them superior to other sectors in certain societal fields. Vaillancourt Rosenau’s [18] review of the literature suggests that private for-profit actors are creative and dynamic, innovative, able to adapt to rapid changes, good at replicating successful practices and at performing complex tasks, while public organisations are better in fields such as regulation and policy management as well as in ensuring equity, securing public interest and preventing discrimination or exploitation. Finally, it has been suggested that not-for-profit organisations are those who express compassion and commitment to individuals and are concerned with moral codes and individual responsibilities. Not-for-profit organisations have often been seen as a group of organisations, which base their actions on certain ideological or religious commitments [19] and which are able to meet the social need that the state and the market are unable or unwilling to satisfy [20]. Third sector organisations have been instrumental in developing the services that presently form the basis of the western welfare states e.g. [21] and provide a major part of health and social services especially in countries with social health insurance [20]. However, as to actual contracting out of health care and social services, the literature mainly discusses the relationship between the public sector and for-profit organisations.

The arguments for contracting out often include beliefs in improved cost-control and more flexible organisation [22], improved resource allocation and better management [2], cost-efficiency and better service quality [11] as well as willingness to concentrate on the ‘core service’ of the organisation e.g. [8,9,11,22-24]. It has often been suggested that in the public sector there is willingness to benefit from competencies and technologies applied by private providers, a desire to import additional resources in the public sector as well as a belief that private actors are able to operate more efficiently [2,6,9,25]. Furthermore, it has been reported that improvements in quality, increased user satisfaction, a way to motivate employees and a wish to reduce the scope of the state are the driving motivations for increasing the scope of private sector service delivery [6]. However, the literature also suggests that compared to their public or not-for-profit counterparts, private for-profit providers fare poorer in terms of e.g. service quality [26], staff density [27], psychosocial working conditions [28] and costs of care [29]. Contracting with the public sector is also suggested to undermine the terms and conditions of employment [30] as well as to create an unstable environment in which organisations are no longer able to offer secure long-term employment for their employees [19]. The research evidence on the performance of public and private providers is, however, controversial and reverse results have also been reported e.g. [27-29].

As to the state of democracy and citizen involvement, contracting with the private sector has raised several concerns. Hodge and Coghill [3] state that privatization of service provision undermines political accountability but increases the importance of managerial and market accountability. In other words, through increased contracting, the power relations between societal actors may alter and the democratic state may incrementally change towards a more corporatist one [25]. Warner [29] goes so far as to argue that the movement from the public sector to the market diminishes the room for citizen involvement, which may be seen as a key to democracy. Flinders [31] sees privatization policies as a “Faustian Bargain” and suggests that while some short-term efficiency improvements and costs savings may be gained, the different privatization policies are likely to result in substantial political and democratic costs. Finally, it has been claimed that competition, which is often involved as services are contracted out, provides a poor foundation for equity between citizens [25,29].

As to the provision of health and social services in particular, Vining and Globerman ([32], 79) suggest that the criticisms of contracting out concern at least the following issues. Firstly, in the area of health care and social services the competition is often limited, leaving the purchasers fairly vulnerable to opportunistic behaviour, such as overcharging for services. Secondly, the complex nature of health and social services poses challenges for definitions of best quality as well as for monitoring provider performance. Finally, contracting involves a risk of poor performance but not necessarily a possibility to cancel the contract. In the empirical literature the reluctance has related especially to the special nature of health care and social services that often include regulatory tasks, prevention and ability to react in a case on a crisis [8,23].

### Research context and methods

Municipalities are responsible for organising health care and social services for their residents in Finland. The municipalities have been free to contract with private providers since 1984 in social services and since 1993 in health care services. However, due to a deep recession in 1990s the issue of contracting did not become topical in the local health and social policy until the early 2000s. Since then the municipalities have expressed a growing interest in contracting out their services with private for-profit and not-for-profit providers.

Municipalities are currently in a process of reorganising the governance of their service structures in Finland [33]. One of the developments has been to reorganise their services by introducing a purchaser-provider split in the municipal organisation [34]. In addition, the new capacity needed to meet the growing demand of sheltered housing and home help for the older people is mostly purchased from the private sector.

A significant part of the housing services provided by private sector has traditionally been provided by private not-for-profit providers, with which the municipalities have already co-operated for decades. However, changes in the legislative environment (e.g. EU competition law) have made the contracting with private providers a process emphasising competition instead of co-operation. In addition, for-profit providers have been increasingly interested in the growing market of elderly care services. These developments have altered the positions of not-for-profit providers that now are forced to compete on the municipal contracts with for-profit providers. In 2009 the market shares in sheltered housing were at macro level 46%, 32% and 23% for the municipalities, for-profit providers and not-for-profit providers respectively [35]. However, the proportions vary locally.

In primary health care the volume provided by private providers is smaller compared to the care for the elderly [35]. However, the share of the private sector has been in increase since the mid 1990s. The total volume of primary health care services purchased from the private sector increased from 28 million Euros in 1995 to 154 million Euros in 2008. In 2009 there were 37 outsourced health centres in Finland, which served some 7% of the Finnish population [35]. In addition, especially the municipalities in rural areas have experienced difficulties in recruiting physicians to their health centres. This has opened a new market niche for private for-profit recruitment agencies that deliver physician and nursing workforce for health centres that struggle with recruitment problems. Also out-of hours A&E services are often purchased from private sector due to the recruitment problems. In primary health care the services purchased form private sector are mostly provided by for-profit providers.

Despite of the growing interest in contracting with private providers in Finland and elsewhere, only few studies have explored how policy makers ground their decisions. We try to fill in this gap by studying the framings the local authorities use as they talk about contracting out of health care and social services. In the analysis we used frame analysis, the method initially introduced by Goffman [13]. The way a certain policy problem is framed is important as framing creates rationales that authorize some policy solutions and not others [14]. Frame analysis provides a tool for revealing these rationales. It also enables us to uncover the underlying beliefs, perceptions and appreciations of the policy makers [15]. Finally, it provides a tool to depict and engage the array of arguments and their counter arguments that encircle complex and controversial policy issues that are characteristic of health care and social services [36].

We use interview data drawn from six municipalities in Finland. Of the participating cities, four are included among the ten largest cities in Finland and altogether they represent circa one-fifth of the Finnish population. The study is part of a larger research project exploring the separation of purchasing and provision functions in *primary health care and elderly care* services in Finland. The research plan has been written according to the guidelines of The National Advisory Board on Research Ethics. The selection criterion for municipalities was their administrative structure: the participating municipalities were selected from the municipalities that have separated purchasing and provision functions in their health care and social service organisations. The six municipalities participating in this study were selected because they represent different geographical areas in Finland [south, west, and north] and because they are in different stages in the process of separating purchasing and provision. All of these municipalities have also outsourced some of their services to for-profit and not-for-profit organisations.

The interviewees include civil servants and elected officials. Of the c*ivil servants* the researchers chose to interview those responsible for purchasing health and social services for the citizens. They play a crucial role when the political decisions are prepared for the city council. *Of the elected officials* the researcher chose to interview those who are responsible for setting the annual budget for health and social care and for the political decisions on purchasing services from private providers. The data were collected through group interviews (n = 13) with 2–6 participants. In addition, four interviews with only one participant were conducted. The interviews were organised separately for civil servants and elected officials. According to the principles of the National Advisory Board on Research Ethics in Finland a study like the one at hand is exempt from requiring ethics approval.

In all the interviews a thematic interview form concerning the purchasing practices of the municipality was applied. The interviewees were asked directly about their reasoning for purchasing services from the private sector. However, the interviewees referred to contracting issues also elsewhere in the interviews. The interviews were taped and transcribed by five research assistants. All the research assistants were asked to sign a written consent for professional secrecy.

The analysis was conducted in three phases (Table 1). The first author conducted the analysis and participated in the data collection with the two other authors. The final interpretations of the results were discussed among all the authors. In the analyses it was acknowledged that the interviewees could use several argumentation frames within a single interview. However, the purpose of the analyses was to explore the argumentation frames in general and not the argumentation of a single interviewee. The results are reported following this principle.

**Table 1:** **Three phases of the analysis and their results (Adapted from Gamson&Lasch**[37]**: 399–400)**

	**First phase**	**Second phase**	**Third phase**
**Actions**	Data reading and grouping of statements according to their content	Completion of”signature matrix”	Aggregation of the initial frames into five final frames
**Results**	Eight initial frames*-Strategic planning and rational decision-making- Irrational decision-making- Municipal economy- Market orientation- Citizens’ best- Benchmarking- Fire fighting- Exogenous motivations*	Descriptions for each initial frame with the help of signature elements (Table 2).	The final frames*: Rational reasoning, Pragmatic realism, Promoting diversity among providers, Good for the municipality, Good for the local people*

In the first phase of the analysis, all the references to contracting with the private sector were extracted inductively from the data and grouped according to their content. This resulted in eight contentually consistent ensembles, i.e. initial frames. In the second phase a “signature matrix” drawn from the work of Gamson and Lasch [37] was employed (see also [36]). In their work Gamson and Lasch ([37], 399–400) suggest that every frame has certain “signature elements” that help to reveal its core and position. These elements include *metaphors, exemplars, catchphrases, depictions, roots, consequences* and *appeals to principle*. These elements were employed in order to describe the eight ensembles established in the first phase. As a result of the second phase a signature matrix was completed (Table 2). Finally, analysing the signature elements of the initial frames, the eight initial frames were then aggregated into five final frames.

**Table 2:** Examples of a completed signature matrix for two initial frames: Citizens’ best” and “Fire fighting”

	**Citizens’ best**	**Fire fighting**
**Metaphors**	“Shopping around in the market place of health care and social services”; “Choosing services as one chooses the toppings for one’s pizza”	A municipality as a “fire fighter” extinguishing fires here and there
**Exemplars**	***Municipality:*** Enabling choice and citizen involvement; taking care of citizens; creating continuity of care*** Private providers:*** Providing something more than the public sector; meeting diverse citizen needs; enabling choice and personalised services	***Municipality:*** Trying to ensure the availability of services; object rather than active subject*** Private providers:*** Able to meet the acute needs of the municipality e.g. delivering workforce.
**Catchphrases**	Citizens’ right to choose their own provider and make their own decisions; representing the will of the citizens; individually tailor-made services; taking care of our citizens; continuity of care	Physician shortage; Shortage of facilities
**Depictions**	The decisions are based on the citizens’ best. Privatizing provides citizens with better opportunities to choose their provider and with a more diverse selection of providers. On the other hand, the decision not to privatize is based on a notion that public sector needs to take care of citizens and that market forces endanger equity and equality.	The decision to privatize is argued with acute needs e.g. acute physician shortage. The decisions are made on a case-by-case basis.
**Roots**	Public choice; Paternalism; Individualism	Physician shortage; lack of monetary resources; service needs
**Consequences**	***Positive:*** Citizens get what they want; tailor-made services*** Negative:*** Failed monitoring may endanger the quality; patient safety may suffer if private providers employ staff that is not familiar with the area and local conditions	***Positive:*** Availability of services & better access***Negative:*** Short-term improvements only, privatization as an emergency solution
**Appeals to principle**	The goal is to work for the best of the citizens: increased private provision enhances the ability to make choices	The goal is to ensure that the services are available even though there is a shortage of resources etc.

## Results

In this section we present the results of the frame analysis, which resulted in five frames concerning the grounds for the decisions. Summaries of the frames and data extracts for each frame are provided in Table 3.

**Table 3:** How the justification to contract out/not to contract out is formulated and what is the interviewee position in each frame, description and data extracts

	**“Rational” reasoning**	**Pragmatic realism**	**Promoting diversity of the providers**	**Benefits for the municipality**	**Good for the local people**
**Justification to contract out**	The decision to contract out is a rational decision based on strategic planning and careful considerations taking into account the good of the municipality as a whole.	The decision makers are forced to choose an alternative, which from their point of view, is suboptimal or undesirable, but which is the only possible alternative in the present situation.	Outsourcing is a means to create provider diversity in order to improve quality and efficiency, gain cost-savings and create benchmark for public providers. In addition, diversity is seen as a source of flexibility and citizen choice.	Outsourcing is seen as a tool to boost municipal economy through job creation and increased tax revenue.	Outsourcing is seen as a means to ensure high quality, tailor-made services and to provide choice for the citizens.
**Example**	*“The planning takes into account the whole range of services, what there is available in the area and what we might possibly need more. Is it worthwhile to renovate the long-term hospitals that have been transferred to us and to what extent, how much we could preserve in the hospital area and how much it will cost or whether there’s the option of abandoning the service and buying it. I mean we are planning all the time and this is one of the manifestations, we try to look at different areas of Helsinki for what the situation is.”* (Civil servant)	*”We have this chronic problem that our older people are in completely wrong places, in wards in the regional hospital or health centres, and this will also lead to a fairly rapid institutionalisation of elderly patients. In a way the A&E department is a strategic key process used to direct older people with many illnesses into the orbit of specialized health care. And as we are dealing with people who do not know the services and as the municipality is ‘saving’, so to speak, and the A&E services are cheaper when contracted out. But we will get the bill through specialized health care and institutional care for older people.”* (Civil servant)	*”Our model of multiple providers is a way of benchmarking our own provision against another provider to see whether there are new ways to provide services. I must admit though that this public system is pretty rigid in terms of reforms. In a way we must get some evidence that the work can surely be done in some other way.”* (Civil servant)*“The more actors, the more there will be different ways of doing things. I have swallowed the idea that in the future the only possibility in the social services field is to increase productivity at work. Competition is the thing that increases it, especially if you have small units, they will do things differently and they all try to work towards more efficient solutions. But as I said earlier we should be able to create such quality indicators that we could look at it not only from a purely economic viewpoint.”* (Politician)	*“And it started to appear right from the start as a local employment scheme, which it is to the greatest extent. And as we’ve got high unemployment numbers in the area and high structural unemployment, we’ve always had it, there’s been very little discussion about it but it’s the thing that’s continuing to cause pressure in the background, that we should organise our operations in a way that would make visible our local employment and local need for jobs and to design systems that support local initiative.”* (Civil servant)	*”It probably is this”ageing in place”, the notion that the services somehow come closer to people, in the form of individualised solutions. The role of the municipality will become stronger in case management guidance, the role of the public sector as a promoter of solutions and as information and guidance services. And there will be countless ways to provide services and people will try to find solutions that suit them best and they will also pay for them.”*(Civil servant)
**Justification not to contract out**	The decision not to contract out is based on the view that there are certain core services, which the municipality is willing to preserve. The costs of out-sourcing are seen excessive compared to the perceived benefits.	The decision makers are forced to choose an alternative, which from their point of view, is suboptimal or undesirable, but which is the only possible alternative in the current environment.	In relation to the aim of the diversity, a certain amount of public provision should also be preserved.If there is excessive diversity the chances are that the service system becomes too fragmented and the coordination of the system may become difficult resulting in inefficiencies and extra costs.	Not to contract out is seen as a tool to prevent multinational companies from obtaining a local monopoly and ruling the small local firms out of the market.Public sector wants to preserve its role as a good employer employing people in the area.	It is seen that citizens should be protected from the market forces. There is willingness to ensure the quality and continuity of care.
**Example**	*”Well certain official services should not be contracted out at the moment, at least not at the present moment, in the sense of responsibilities and other things. But we’ve seen some things, kind of strategic issues, too, that the city provides and operates. I don’t think it’s sensible to contract out all areas in our health care and social services field.”* (Politician)	”*We had a situation in C., I believe, there was a health centre which could not get a doctor for two years. We wanted to buy them physician services so that K. would get a doctor. They turned it down for ideological reasons because they think it will lead to inequality. Or I don’t know what the reason was but the plan fell through.”* (Politician)	*”And in our elderly services, how much did E. say, it’s quite preposterous what they’ve purchased. Fifty-four percent of the housing services. There actually is no room for further increase there. The municipality should have operations of its own to be able to benchmark.” (*Politician)	*“It would be different if the actor in elderly services is a local business because we support local business activities. But the business in question that organises the physician services for the health center is a listed company and owned by foreigners, I believe. The tax collected from our municipal residents is not meant to be used for this but the activities should benefit the whole region, the local businesses, and we do not have such health care businesses.”*(Politician)	*”These are the resources that we are actually measuring. But you certainly need to have specific terms and conditions as the extreme horror scenario would be to return to the period of auctioned paupers, someone will make the lowest bid and the quality will start to suffer and our elderly citizens will be left without food. It’s just like wading through a quagmire you should have clear tools for measuring the quality. On no account should we leave them at the mercy of the market forces.”* (Civil servant)
**Interviewee position**	A rational actor who tries to defend the rationality of the decisions and to promote a comprehensive decision-making process despite external pressures influencing the decisions.	Rational actor forced to adopt a pragmatic and realistic position towards the decisions as a means to adapt to prevailing environment	An actor who is willing to create diversity of providers and alternatives for citizens as long as it improves providers’ operational measures and does not endanger public provision	An actor who does what is best for the municipality as a whole.	A actor who bases their decision on the notion of “the best of citizens”

### “Rational” reasoning

In this frame, the decisions to or not to contract out the services were represented as resulting from rational comprehensive decision-making processes. The decisions were represented as being based on strategic and rational planning and careful considerations that take into account the strategy of the municipality as a whole. The arguments for and against contracting were often something like *“we look at the big picture and then decide what is the most appropriate way to organise the services”.* Moreover, a fairly common viewpoint was that there are certain ‘core services’ that the municipality wants to preserve or which were even seen as compulsory for the public sector to carry out. This applies especially to health care. Contracting out was, thus, seen as a tool to organise services that are not included among these ‘core services’. All in all, at first glance it seemed that were no ideological or personal preferences guiding the decision-making.

However, while the decisions were argued through objective, often financial or strategic measures, there were references suggesting that these arguments were partly used as rhetoric tools to convince the interviewers or in order to veil other potential arguments for the decision. Thus, while the initial grounds seemed to be fairly strategic and rational, the actual actions appeared to be influenced by several external factors. In the example below, for instance, the process is described as being rational while in actual practice it seems to be fairly incremental.

"”Well as a matter of fact a lot has happened in different projects, they’ve produced quite a lot of the city’s operations, some of them as permanent purchased services. We’ve had those, but I don’t think it’s going to work in actual practice […] I mean we’ve always kind of started from the growth of service needs, or some other reason.” Civil servant"

Furthermore, several interviewees referred to a municipal strategy as a basis for their decisions. However, in some interviews it appeared that while the decisions were framed as strategic, in practice there was no actual strategy for the organisation of the services or it was potentially influenced by relative political strengths. The following quote is presented to illustrate the situation:

"“I think it’s better for us to buy strategic and clearer entities (…), it’s sensible for both the client and municipal economy that the actor who is responsible does so as comprehensively as possible. And then again it may be that when contracting out a field, for instance, if we are speaking in a competitive sense, we need some leeway; while we would and surely will be taking specific owner alignment measures as to which of these are the strategic entities that we will hold on to.” Politician T"

In general, the interviewees positioned themselves as rational actors who try to defend the rationality of the decision-making processes in spite of several factors that actually influence the decisions or actual implementation of the policies.

### Pragmatic realism

In this frame the decision to contract out or not was described as resulting from a situation in which the politicians or the civil servants implementing the policies are put “between a rock and a hard place”. That is, they are forced to choose between two or more – from their point of view – unsatisfactory or suboptimal alternatives, such as choosing between contracting out to the private sector and compromising service availability. This applies especially to politicians that are responsible for the contracting out decisions.

Especially the civil servants described situations in which they had to choose an alternative, which – again from their point of view – is suboptimal or undesirable, but which is the only possible alternative in the current environment. For instance, there were situations in which an interviewee was reluctant to contract with a private provider but was forced to do so because of a lack of physicians, facilities or other resources and because the politicians saw contracting as the best policy option. Politicians, in turn, described situations in which they would have wanted to contract out a certain set of services, but “the political opposition they faced was so substantial that it was impossible”.

In this frame the interviewees portrayed themselves as actors whose rational actions are restricted by the circumstances created by the political environment and by other exogenous factors influencing the policy decisions, such as a lack of resources, past decisions and legislation. The main undertone in the interviewees’ talk was that they *“do what a man’s got to do”*. That they act rationally in a less rational decision-making process and adapt reluctantly to the prevailing situation.

### Promoting diversity among providers

In this frame contracting out was described as a means to increase the number of providers delivering heath care and social services and to create diversity among them. It was believed that diversity is beneficial as it creates competition between providers and enhances innovation, all of which are believed to result in improved quality, efficiency and cost-savings. Furthermore, the interviewees were willing to create a benchmark for public provision. It was thought that private providers possess certain qualities, which make them superior to public providers in terms of efficiency, cost-effectiveness and quality. Moreover, it was stated that cooperation with private providers is easier than with the municipality’s own providers as the private providers “do what is agreed upon and do not show up in the middle of the contract period to beg for more money as the public providers might”. It was also thought that increasing the number of providers would provide citizens with more opportunities to choose a provider and to receive personalised services. Finally, diversity was seen as a source of flexibility, which protects the service system against sudden changes potentially occurring in the future. Relating to this, there were references to the idea that diversity might enable the municipality to focus on its core tasks letting the private sector to take care of the services outside of this very core. A civil servant described the situation as follows:

"”(…) perhaps there’s the idea that the focus is on sheltered housing which is actually a sort of market-driven field nationally, but the city made a decision in the 2000s that the market is working pretty well, so we’ve basically making an effort to seek growth and to focus on our core operations.” Civil servant"

However, several interviewees also stated that*”not everything should be contracted out”*. They felt that diversity also means the existence of a certain amount of public provision. This was seen crucial also from the benchmarking point of view, as the decision makers should be able to evaluate the performance of private providers against that of public providers. In addition, too much diversity could mean that the service system may become too fragmented and the coordination of the system as a whole might become difficult resulting in inefficiencies and extra costs.

### Benefits for the whole municipality

In the fourth frame contracting out was described as a means to boost the economy and the employment rate of the municipality. On the one hand, purchasing services from local private providers was seen as a tool to create jobs and to support employment in the area. On the other hand, it was seen as a means to increase the municipal tax-revenue as the local firms are subject to a community tax collected by the municipalities. However, this argument was used mostly as a conditional one: Contracting out was seen as an option only if it was possible to purchase services from local providers. One of the interviewees described the matter as follows:

"“It’s a rather dominant opinion at the moment that we should try to attract business activities in this field and to make it more diverse. But since we know from bitter experience that if a purchasing decision is made, a multinational company owned by a foreign pension fund will come along and buy it and polish their operations to perfection while we are left practically empty-handed. We’ve had the same disappointments here as elsewhere in Finland. In effect, our economic development office is trying to figure out how to keep the people in the hands of businesses with a human face.”Politician"

In this frame, the decision not to contract out was also related to the quote above. The main argument against contracting with private providers was that there would be a danger that big multinational investment companies would come and attain a local monopoly in service delivery. This, in turn, would result in ruling the small local firms out of the market. In addition, the local authorities expressed their willingness to employ local people to preserve their image as a good employer. This all related especially to elderly care services.

In general, the interviewees portrayed themselves, as ones who make the decisions that they think are best for the municipality and its economy as a whole. In this sense the frame approaches the”rational” reasoning frame in which the good of the whole municipality was also considered. However, in this frame the arguments relate clearly to the municipal economy and employment of the area, while in the”rational” reasoning frame the descriptions of what is good for the municipality are focused more on health care and social services and described in a more abstract manner.

### Good for the local people

The ‘good for the local people’ frame was the only frame in which citizens were considered as the first priority. Contracting out was seen as a means to ensure high quality services for the local people. On the one hand, contracting with private providers was seen as a means to ensure that citizens will get high quality services also in the future. A common argument was that *“we are not going to survive alone in the future, but need private providers to help us to meet the growing service needs”.* On the other hand, the argument was more qualitative: The local authorities saw contracting with private providers as a tool to ensure that citizens are able to choose among different service providers and acquire “high-quality” and “personalised” services. This argument was based on the idea that in the future the role of citizens will alter from a patient or client towards an active consumer who *“shops around in the service marketplace”*. This argument was also used to justify pure privatization or at least increasing co-payments for services through the introduction of vouchers.

"”Well it’s a question about money too and that’s why we also try to make a conscious effort to reduce the city’s expenses since the voucher is actually never fully commensurable with the cost of the service. So as to vouchers the city’s share compared to a service provided by the city will be lower. Issues such as this are also at stake.”Civil servant"

The decision *not* to contract the services out was, in turn, based on the countering view. It was thought that it is the duty of the local authorities to *“protect citizens from market forces”*, especially when vulnerable patient groups and old people were concerned. It was also seen that contracting out would not guarantee continuity of care as staff turnover was considered higher among private providers that in the public sector. Finally, it was thought that as the measures to monitor the quality of care are fairly poor, the guarantee of care quality could be endangered if the services were contracted out.

## Discussion

The analysis resulted in five frames, which the local politicians and civil servants interviewed in this study applied to describe their decisions on contracting out of health care and social services. The insights did not differ considerable between the civil servants and local politicians. There were arguments for and against contracting out in each stakeholder group and in each frame arguments from both civil servants and local politicians.

The decisions were framed in five ways. Firstly, the interviewees portrayed the decisions as rational and free from political, ideological or other exogenous influences. Occasionally, however, the use of rational descriptions rather resembled a rhetoric tool than the actual grounds for the decisions. This finding lends support to the study by Stold and Winbland [6] suggesting that while the decision-making process leading to contracting with the private sector seems to include e.g. economic arguments there are also ideological factors as well as elements form policy diffusion that guide the decisions. As a whole it seemed that the interviewees were aware that it might be more reasonable to argue the decisions on contracting with the private sector through strategic grounds rather than revealing personal preferences of the issue.

In the first frame several interviewees mentioned that there are certain ‘core services’ that the local authorities are not willing to contract out. Argumentation through ‘core services’ has also appeared in previous studies on outsourcing and contracting out but the consensus on the content of these services has remained elusive (e.g. [9,11,22,24,31]). Our data suggest that the ‘core services’ would include at least preventive and regulatory services (compare [8,23]). In general, however, the core services were rather vaguely defined also in this study and might be an interesting subject for further research.

In the second frame the interviewees described situations in which they were forced to choose an alternative which, from their point of view, was suboptimal or undesirable but which was the only possible alternative in the present situation. In this frame, the interviewees admit that the decision-making process is not rational and that they try to adapt to it even though they were not supportive towards the final decisions. Thus, the actual decisions are trade-offs between different values and interests, and the decision makers are not always able to make decisions that would be accordance with their own values nor with the best of the local people. Several interviewees reported situations in which the decisions, potentially most beneficial for citizens, were not implemented, as there were other more important objectives that were pursued at the time. This leaves us to contemplate if contracting out really is a “Faustian Bargain” [31] through which the policy makers are able to gain short-term efficiency improvements and costs savings, but which long-term results in political and democratic costs, because the best of the local people is has not been the point of the departure as the decisions are made.

These decisions potentially suboptimal from the citizens’ point of view are often influenced by the institutional settings and cultural contexts, as well as by individual beliefs and ideologies [11]. In our data probably the most influential factor affecting the decisions to contract out was the administrative structures of the municipalities. All the municipalities had adopted purchaser-providers split in their organisation, which had directed the municipalities already in the path involving the aim of increasing contracting out *per se*.

Thirdly, contracting out was justified through the willingness to promote service provider diversity, which was believed to result in improvements in public service provision and in increased opportunities for citizen choice. The improvements in public provision were seen especially resulting from increased competition and benchmarking opportunities with private providers. These, in turn, were believed to lead to improved quality of care and efficiency in service delivery in the public sector. This rationale seems to be in line with the arguments reported in previous studies on outsourcing and contracting out (e.g. [2,25]) as well as with the literature addressing the properties of different ownership types [18].

However, there are also studies that do not support these fairly stereotypical distinctions often presented in the literature. The findings of a recent study by Stolt and colleagues [27], for instance, did not support the notion of public providers learning from private providers. Rather, the quality of care in public units seemed to remain constant irrespective of the rate of competition between the providers in the area. In addition, studies by Warner [29] and by Comondore and colleagues [26] do not lend strong support to performance improvements of contracting in terms of quality and cost-savings (e.g. [26,29]). There have even been cases in which the evaluation of the performance of private providers has been significantly hindered, as the contract documents have not been available for the public [38]. The concerns of the transparency and the ability to monitor private providers’ performance were expressed also in our interviewees (see also [32]).

The fourth frame was based on the aim to boost municipal economy through job creation and increased tax revenue. The interviewees were mainly willing to contract with the private sector only if it meant purchasing services from local, often third sector providers. Thus, the prevalent opinion was that the multinational for-profit companies would not be the most desirable partners due to their relative market strength compared to the small local providers. There seemed to be a real concern among the interviewees that they are not able to preserve local service provision due to the current competition law dictating that public procurement procedures be applied to purchases exceeding 100 000 Euros. Other scholars have also expressed their concerns about the effect of competitive tendering procedures on especially third sector organisations (e.g. [39]). Several interviewees also mentioned that they are willing to preserve jobs in the public sector and thus, preserve their reputation as a good and responsible employer. Similar arguments have also been reported elsewhere [9,24].

The fifth frame was the only frame in which citizens’ best was applied as a point of departure. In the other frames the arguments for and against contracting out were mainly related to the benefits the municipality would potentially gain trough contracting. It could be argued that the improvements in the municipal economy and the cost-savings gained through competition, for instance, would in the end also benefit the local people. Potentially this is the case. However, the reasoning for the existence of the public organisations and the legitimacy of the decision-making authorities are based on the notion of them serving the local people. The public provision and the monopoly status given to the public sector in certain service fields have been justified through the importance of the product and the protection of vulnerable client groups (e.g. [18]). The needs of patients in the context of primary care and elderly care are inherently complex and require cooperation between several societal sectors and promotion of integrated care. Successful integration of health care and social services especially in the care of elderly patients would potentially result in benefits for the patients [40] as well as in reduction of costly hospital admissions [41]. However, the interviewees expressed very little concerns about the consistency of care chains or continuity of care. In the cases these were discussed, the interviewees described situations in which there were other factors that tip the scales in favour of other values than the best of citizens in terms of comprehensive care.

Throughout the interviews it appeared that the orientation towards the role of citizens is changing. This applies especially to elderly care services. Several interviewees saw that the senior citizens are becoming active consumers willing to “shop around” in the market place of health care and social services (compare [29]). In addition, there was also a fairly strong belief that citizens are willing to invest in their services and purchase them also out of pocket. The argumentation focusing on citizens’ best interests was indeed also applied to justify the increasing co-payments that would result from the introduction of vouchers and from restrictions on the eligibility criteria for receipt of services. These developments seem to be somewhat similar than those reported in Sweden [42].

The municipalities included in this study represent large or medium sized cities in Finland and thus, the results cannot be extrapolated to small and rural municipalities. In smaller municipalities the argumentations are potentially fairly different as the provider market is often non-existent, which undermines the feasibility of contracting with the private sector as a policy tool. In addition, small municipalities, often located in rural areas, potentially use contracting for different purposes that do the larger cities. The rural areas in Finland have experienced major difficulties in recruiting physicians in their health centres. Those municipalities have mostly used contracting as a tool to ensure physician services by contracting with recruitment agencies that deliver physician and nursing workforce for health centres that struggle with recruitment problems. The large cities, in turn, have often interest to seek also for benchmarking opportunities and provide the citizens with opportunities to choose among several service providers. The municipalities participating in this study are potentially among the municipalities with the most positive position towards contracting with private providers, because they have adopted market-oriented administrative model also in their own organisation. Thus, the results do not potentially reflect the opinions overall in the country.

The data were collected through group interviews that may influence the way the interviewees talk about contracting with the private sector. However, an effort was made to reduce the barriers to talk about contracting out by organising separate interviews with the civil servants and the politicians. In addition, we have focused only on primary care and elderly care services and thus questions related to secondary care and for instance to elective surgery have not been addressed here. These services are potentially very different from the services discussed here and thus deserve study in their own right. Finally, the cross-sectional study design results in fairly static analysis and results. However, we acknowledge that the frames used to argue different contracting strategies potentially vary over time and a contracting strategy can be argued through several frames even by one interviewee. The results of the study provide the reader with the variety of the argumentation frames which are potentially used to argue different contracting strategies over time.

## Conclusions

This study suggests that the policy makers use a number of grounds to justify their decisions on contracting out. To some extent, the argumentation frames concerning contracting out were also consistent with the findings of earlier studies on contracting out and outsourcing decisions. Most of the arguments were related to the benefits of the municipality rather than on what is best for the local people. The interviewees saw contracting with the private sector as a means to improve the performance of public providers, to improve service quality and efficiency and to boost the local economy. While there were lots of references on the interviewees’ willingness to make decisions that benefit citizens, it seemed that in practice there are other factors that become more important in the actual decision-making situation. This is potentially due to the complex decision-making environment involving several political and ideological viewpoints and different value bases. Indeed, the interviewees described several situations in which they were forced to make a decision they saw suboptimal or non-beneficial for citizens, as it appeared to be the only possible alternative in the contemporary environment.

It seems that the interviewees believe that citizens are willing to become active consumers who will shop around for services and also purchase them out of pocket. The increasing choice of a provider involves many promises but also a number of threats. As the choice increases there is also a danger that the status of citizens not able to make their choices properly deteriorates and their potential to receive services becomes less likely. Thus, if the choice is increased there should also be proper counseling services for citizens in order to ensure their access to services. Moreover, there were some references to the increasing willingness to transfer the costs of care to citizens. If contracting with the private sector also involves introducing novel financing mechanisms such as vouchers, there is a true danger that the co-payments of citizens will increase. This, in turn, could severely endanger the affordability of the services and lead to even more substantial undermining of the welfare state, which already now is coming apart at the seams.

## Competing interests

The authors declare that they have no competing interests.

## Authors’ contributions

LKT, JL and SM have contributed to the planning and date collection. LKT was responsible for drafting the data analysis that was developed in cooperation of JL and SM. LKT was also responsible for drafting the article and developing it after comments by the other authors. All authors read and approved the final manuscript.

## Pre-publication history

The pre-publication history for this paper can be accessed here:

http://www.biomedcentral.com/1472-6963/12/201/prepub
